# One-second-lead earthquake warning and impact assessment at Campi Flegrei

**DOI:** 10.1038/s41598-026-42593-x

**Published:** 2026-04-08

**Authors:** V. Longobardi, S. Colombelli, A. Zollo

**Affiliations:** Department of Physics “Ettore Pancini”, University of Naples “Federico II”, Naples, Italy

**Keywords:** Natural hazards, Solid Earth sciences

## Abstract

Rapid dissemination of earthquake information during seismic crises is crucial for mitigating the impact of earthquakes, particularly in densely populated volcanic areas, such as the Campi Flegrei caldera (Southern Italy). This region is affected by recurrent bradyseism and shallow seismicity, with recent events up to Md 4.6, very well perceived by the local inhabitants and increasing concern among the local community. In such small regions, conventional Earthquake Early Warning (EEW) systems face physical limitations, due to the short epicentral distances and relatively small magnitudes of the earthquakes. Here we propose and offline tested a hybrid, impact-based, on-site EEW system capable of providing rapid estimates of the earthquake size and expected impact (PGV, PGA), within one second after the P-wave detection. In addition, the proposed system extends the classical concept of an on-site EEW approach by introducing the notion of an *area of competence* (AoC): a circular zone surrounding the recording site in which peak ground motion is expected to fall within a user-defined range around the predicted value, and within which local users or infrastructures can effectively benefit from the warning. The proposed methodology is easily transferable to other volcanic or seismically active regions and might represent a step toward low-latency, impact-based earthquake alert systems designed to enhance community resilience.

**Supplementary Information:**

The online version contains supplementary material available at 10.1038/s41598-026-42593-x.

## Introduction

In volcanic and seismic regions, during the occurrence of seismic crises, the timely dissemination of earthquake information can significantly mitigate the earthquake impact by improving emergency safety actions^1^.

A compelling case study is the current seismic crisis at the Campi Flegrei caldera in Southern Italy, where the ongoing volcanic unrest and related moderate size seismicity are raising increasing concern among local population and authorities in the area.

Over the past 75 years, the caldera has experienced repeated episodes of ground instability, known as bradyseism (slow ground uplift and subsidence), documented since the 1950s^2^. In this period, rapid phases of uplift occurred in 1950–52, 1970–72 and 1980–84^3^. Since 2005, a new monotonic uplift episode has been underway, with a notable acceleration in seismicity observed from 2014 onwards^4,5^. During the last two years, the land instability reached high uplift rates of 2–3 cm per month, and it is being accompanied by an intense and frequent seismic activity with the occurrence of several Md 4+ earthquakes originated at shallow depths (3 km) and causing strong ground shaking (up to several tenths of g) in restricted, few km radius, areas around the epicenter^6^ The urbanized area within the Campi Flegrei caldera is densely populated, with several hundred thousand residents^7^. The recent intense seismic activity, particularly the sudden uplift in the Pozzuoli area, has caused damage to infrastructure and buildings, increasing the urgency for enhanced seismic risk mitigation^6^. Automatic and near-real-time monitoring systems are currently in operation^1,8^, but the time required to detect, process, and disseminate the earthquake information (up to several tens of minutes) — especially for the small-moderate magnitude but well perceived earthquakes — remains a critical limitation in real-time emergency management^9,10^.

Since these earthquakes are generally small-to-moderate in magnitude, the area affected by strong shaking is also limited to a few kilometers around the epicenter and comparable with the “blind zone” of the warning systems, that is the area where the strong shaking occurs before the alert is issued.

Because of this physical constraint, the applicability of Earthquake Early Warning (EEW) systems in such a small-extent volcanic region, as the Campi Flegrei area, is limited.

However, despite the inherently limited lead time imposed by the shallow and proximal seismicity of the Campi Flegrei area, this short time window can still support meaningful response actions when the system is framed beyond the concept of classical alert-based early warning. In such a context, earthquake early warning information is not primarily intended to issue pre-arrival alerts before the onset of strong shaking, but rather to trigger rapid, automated mitigation actions whenever feasible. This integrated “earthquake early response” approach is particularly suited to support human-in-the-loop decision-making and operational workflows, including rapid situational awareness for Civil Protection Authorities (CPAs), preliminary assessment of potential damage and prioritization of inspections^11,12^. In addition, the real-time products generated by the system can directly feed post-event assessment procedures, effectively bridging the temporal gap between earthquake detection and the availability of fully processed shakemaps.

Within this framework, the proposed methodology adapts a standard EEW approach to the peculiar seismicity of the Campi Flegrei area, enabling the rapid generation of P-wave–based shaking and potential damage maps to support Civil Protection Authorities (CPAs) and first responders during emergencies.

EEW approaches are broadly classified into source-based and impact-based methods^1^. Source-based EEW systems rapidly estimate earthquake source parameters (e.g. location and magnitude) to predict ground shaking at distant sites before the arrival of damaging waves. While effective in tectonic regions with larger, longer-duration earthquakes^13–16^, this approach faces severe limitations in areas like Campi Flegrei where seismic events are typically low-to-moderate in magnitude, very shallow, and short in duration^6^.

Impact-based methods bypass the need for full source characterization^17,18^. They rely directly on ground motion observations at individual stations to estimate the potential earthquake impact in real time. Here, we propose a hybrid source/impact-based on-site EEW methodology^19^, to the Campi Flegrei case-study. The early P-displacement signal is used to predict peak ground motion at the site, while at the same time an approximate estimation of the magnitude is obtained to characterize the event size. We calibrate and test the system using a large dataset of earthquakes recorded during the recent unrest at Campi Flegrei. Additionally, show the example of its performance considering two significant earthquake scenarios: the Md 4.4 event of May 20, 2024, and the Md 4.6 event of March 13, 2025.

## Results

**Fig. 1:**
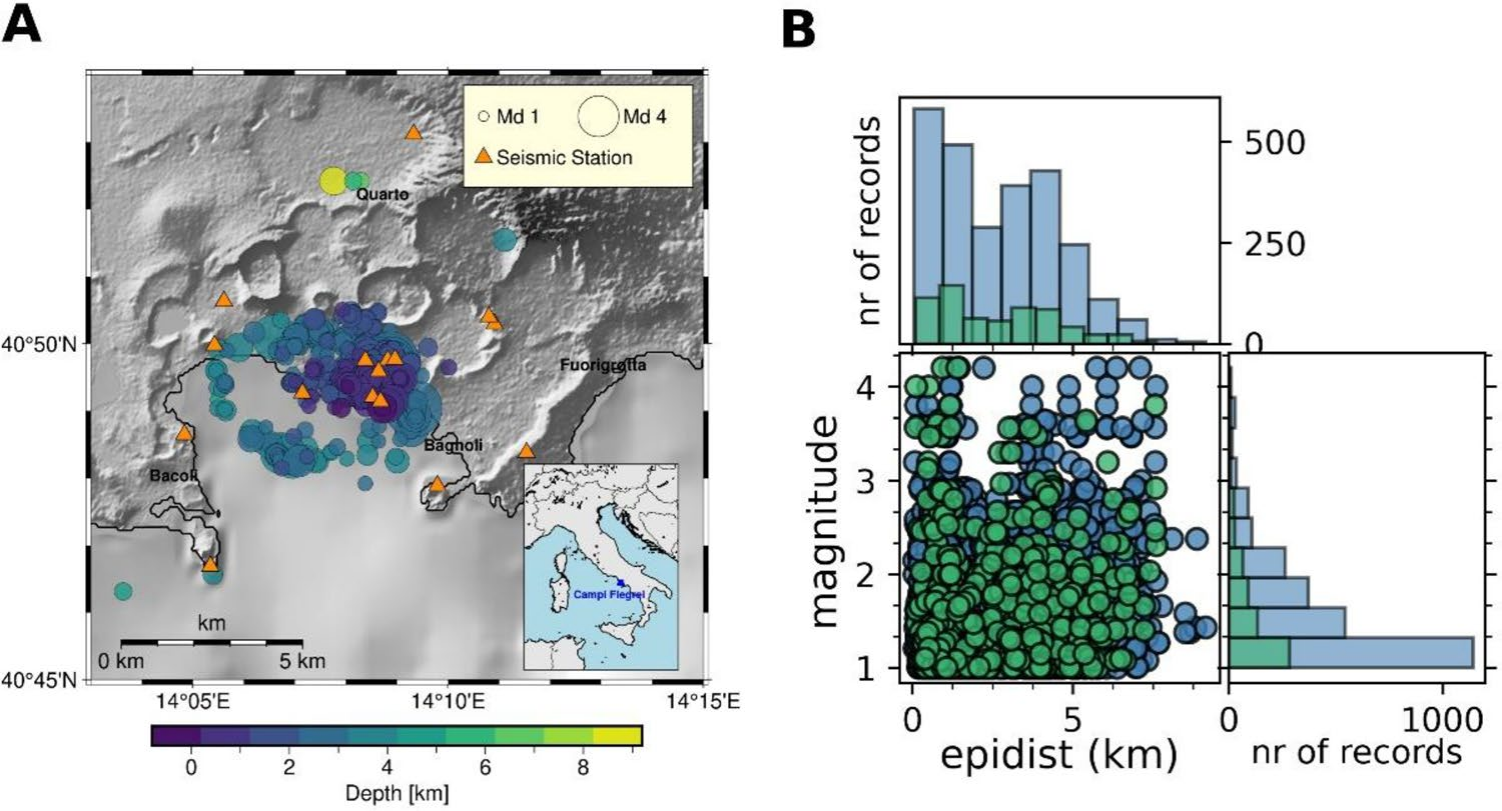
Panel A shows the epicentral location of selected events as well as the position of used stations. As for earthquakes (circles), marker color follows the event depth and marker size follows the event magnitude (duration magnitude Md). Dark orange triangles are seismic stations. Panel B shows the distribution in magnitude and epicentral distance of waveforms from the calibration dataset (blue points and histograms) and from the test dataset (green points and histograms).

To calibrate and test the system we used 700 events with duration magnitude Md between 1 and 4, which occurred during 2016–2024 at Campi Flegrei caldera (panel A of Fig. 1). The preliminary dataset is split into two subsets: the training dataset is used for calibration of empirical relationships, and the test dataset is used for performance evaluation (see panel B of Fig. 1).

We focused here on the estimation, at a single station, with the onsite method of two quantities: the peak ground motion (PGV and PGA) and the earthquake magnitude.

**Fig. 2:**
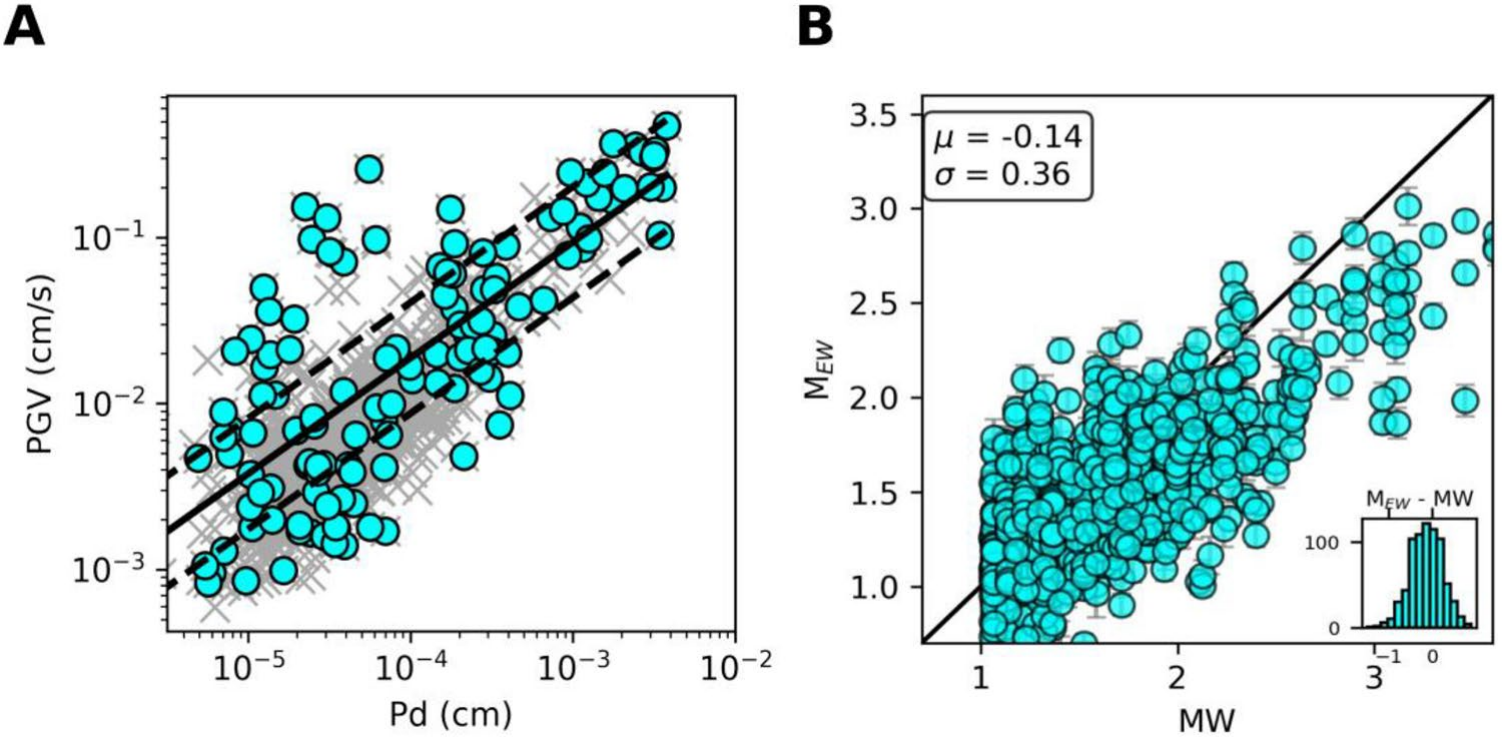
Panel A shows the scaling of PGV vs Pd in a one-second P-time window. Dark grey crosses represent single station measurements on the train dataset. Cyan points represent 2-d binned data (x-bin width = 1 cm and y-bin width = 0.5 cm/s). Dashed lines are the calibrated PGV vs Pd law and its standard error bounds for the 2-d binned data. Panel B shows the single station magnitude estimate from Pd x τ*c* method (M_*EW*_) in one-second P-time window versus observed moment magnitude (MW). Black solid line is the one-to-one MW line. Inset shows the histogram of the difference between M_*EW*_ and MW. Mean and standard deviation of the distribution are reported at top left corner.

As for the PGV prediction, in panel A of Fig. 2 we show the scaling of observed PGV with the P-peak displacement (Pd) measured within a one-second P-wave time window (PTW) for the training dataset. To account for the unbalanced number of earthquakes in the different magnitude classes (panel B of Fig. 1), we calibrate the empirical scaling laws of PGV vs. Pd using 2-D binned data (panel A of Fig. 2). The results of the calibration and the test for the PGA are shown in the supplemental material (Supplementary Figure S2 and S3).

As for magnitude, in panel B of Fig. 2, we present the single-station magnitude estimate for a P-wave time window of 1 s, derived from the seismic moment (M₀). The seismic moment is estimated as the area under a triangular source time function, with Pd as the peak and τ*c* (period parameter) as the duration^20^ (see Data & Methods section). We refer to this magnitude estimate as M_*EW*_. To allow a direct comparison of M_*EW*_, we computed the moment magnitude MW, using the scaling relationship of Iervolino et al. (2024)^7^ that links catalog duration magnitude Md with the moment magnitude MW (panel B in Fig. 2).

The M_*EW*_ method relies on two assumptions: (1) the far-field displacement can be modeled as a triangle with amplitude Pd and duration τc, and (2) the hypocentral distance used in the estimate (Data & Methods section) is a mean hypocentral distance obtained from the distribution of all possible hypocentral distances for the given station (Supplementary Figure S1) determined from the recorded dataset, rather than its exact value.

This latter assumption appears valid for a small area such as the Campi Flegrei caldera, where variations in hypocentral distance are limited due to the distribution of stations and event locations (Fig. 1). Nevertheless, the error on M_*EW*_ estimates is related to the real distance and the empirical distribution of earthquake distances from the concerned station as inferred from the current seismicity. In Supplementary Figure S4, we show the difference between magnitudes computed using point and mean hypocentral distance as a function of the reference moment magnitude (MW) to isolate the effect of the hypocentral distance parameterization. We observe no systematic trend or magnitude-dependent bias over the entire magnitude range considered, indicating that the use of a mean hypocentral distance does not introduce systematic distortions in M_*EW*_ estimates. The prediction error, defined as the difference between M_*EW*_ and moment magnitude (MW), is shown in the inset histogram of panel B of Fig. 2. The average error is centered around zero, with a standard deviation of 0.36 magnitude units.

**Fig. 3:**
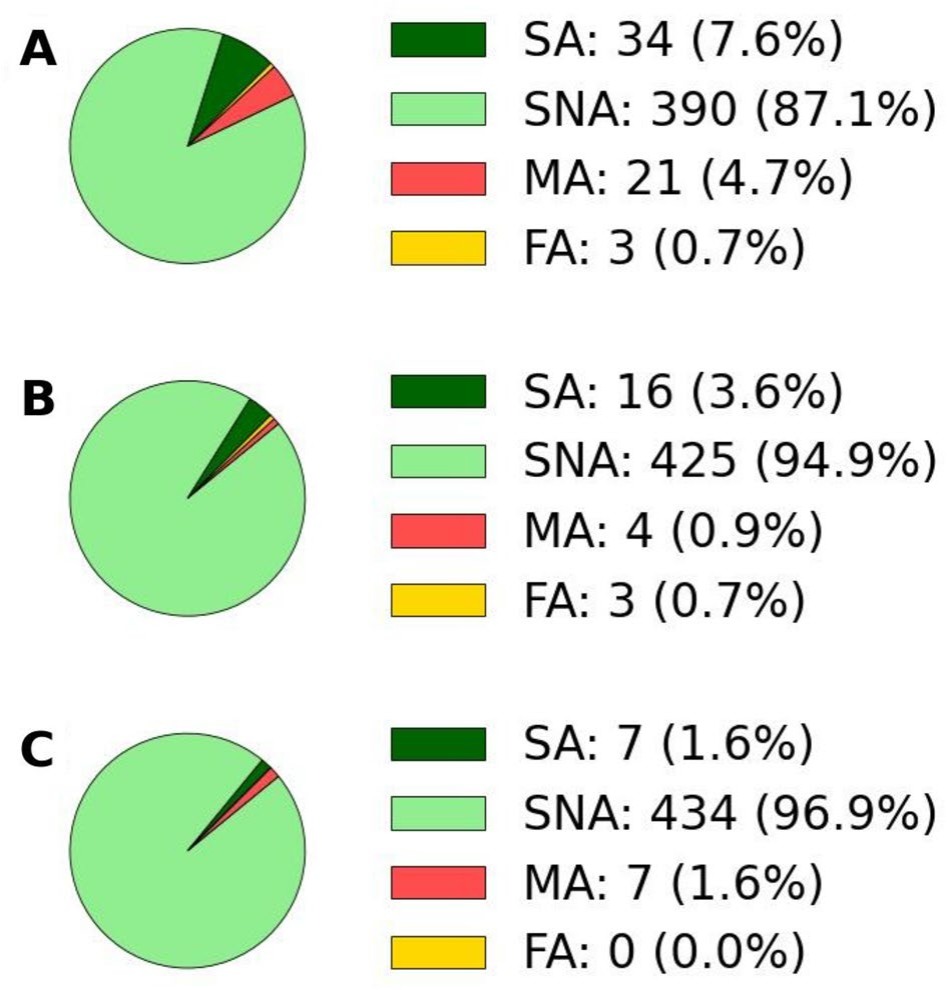
System performance on the test dataset in PTW = 1 s. The performance is assessed in terms of Successful alert (SA) (dark green), Successful No-alert (SNA) (light green), Missed Alert (MA) (red) and False Alert (FA) (yellow) for different thresholds in IMCS. For each IMCS_threshold_, the percentage of SA, SNA, MA, and FA is shown. Panel A shows the performance for IMCS_threshold_ = III; Panel B shows the performance for IMCS_threshold_ = IV; Panel C shows the performance for IMCS_threshold_ = V.

In Fig. 3, we evaluate the alerting performance of the system on the test dataset using different thresholds values on IMCS, from ‘weak shaking’ (IMCS = III) to light damage (IMCS = V). The performance is evaluated in terms of Successful Alert (SA), Successful No Alert (SNA), False Alert (FA) and Missed Alert (MA) and assessed in terms of Precision and Recall (see Data & Methods for more details). In Table 1 we summarized the precision and recall obtained for each threshold.

**Table 1:** Precision and recall of the system evaluated at different IMCS intensity thresholds.

IMCS threshold	Precision (%)	Recall (%)
IMCS ≥ III	91.7	60.0
IMCS ≥ IV	84.2	80.0
IMCS ≥ V	100.0	50.0

At low intensity threshold (IMCS ≥ III), the system exhibits a high precision (91.7%) but a moderate recall (60.0%), indicating that while most issued alerts are correct, a non-negligible fraction of true events producing at least weak shaking are not detected. This behavior reflects a conservative detection capability at low intensities.

At intermediate threshold (IMCS ≥ IV), the system achieves the best trade-off between precision and recall, with precision of 84.2% and recall of 80.0%. This threshold substantially reduces the number of missed alerts compared to IMCS ≥ III, while maintaining an acceptable false-alert rate. From an operational perspective, this balance is particularly relevant, as intensity level IV corresponds to clearly felt shaking, which is likely to generate public concern and may cause minor, non-structural damage.

At high threshold (IMCS ≥ V), precision reaches 100%, meaning that all alerts correspond to true shaking exceedances. However, recall drops to 50.0%, implying that half of the events capable of producing moderate shaking are missed. While such a threshold minimizes false alerts, it significantly compromises the system’s ability to provide timely warnings for potentially impactful events.

**Fig. 4:**
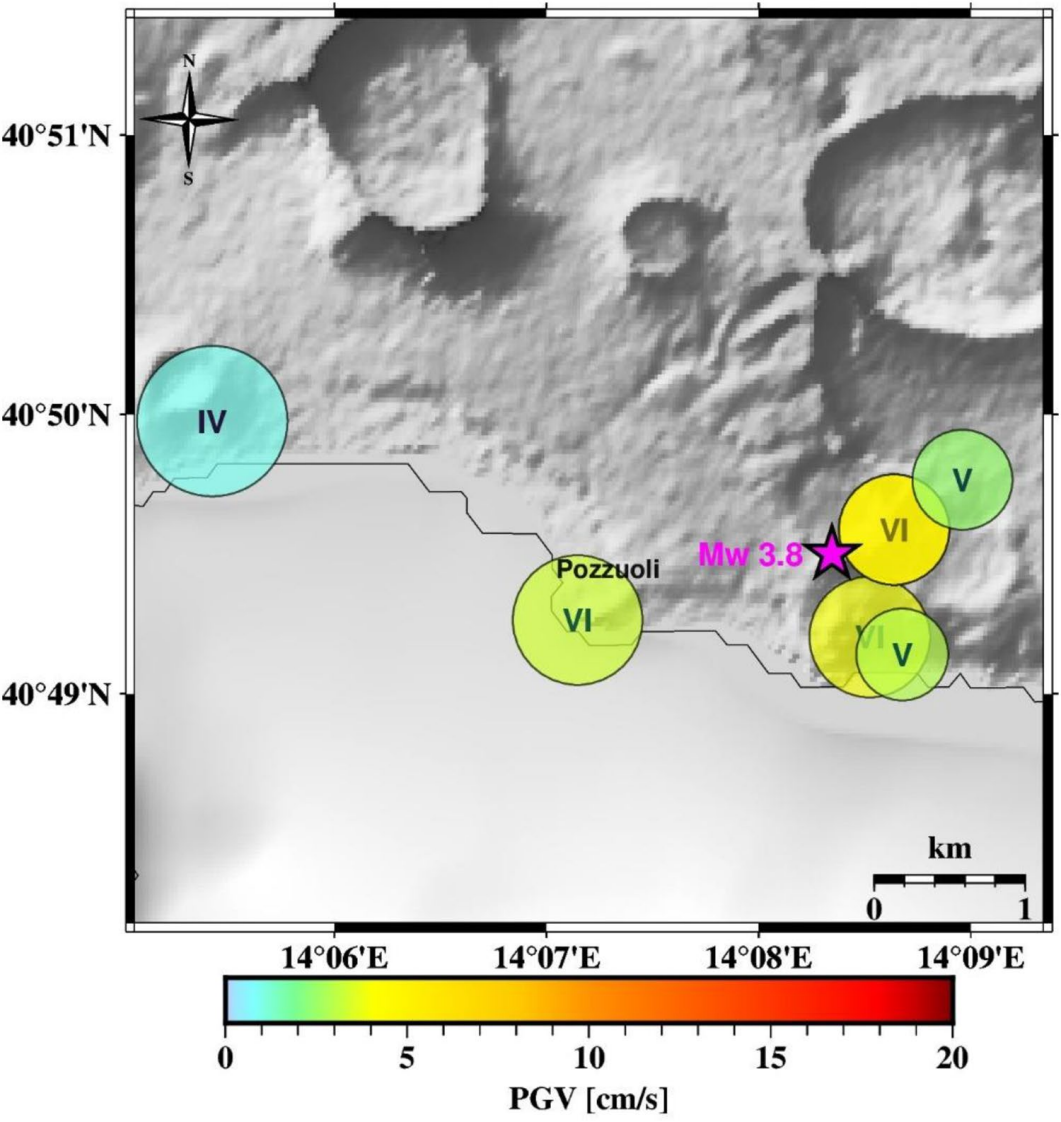
Test on the Md 4.4 earthquake of May 20, 2024. The map shows the result obtained in PTW = 1 s. Magenta star represents the earthquake location (the moment magnitude obtained from Md-MW scaling law by Iervolino et al. 2024 is also shown). Each circle represents the area centered on the station within which the predicted PGV varies between ± 50% of its value. Color follows PGV. In each circle, the estimated shaking intensity (IMCS) at the site is also reported.

**Fig. 5:**
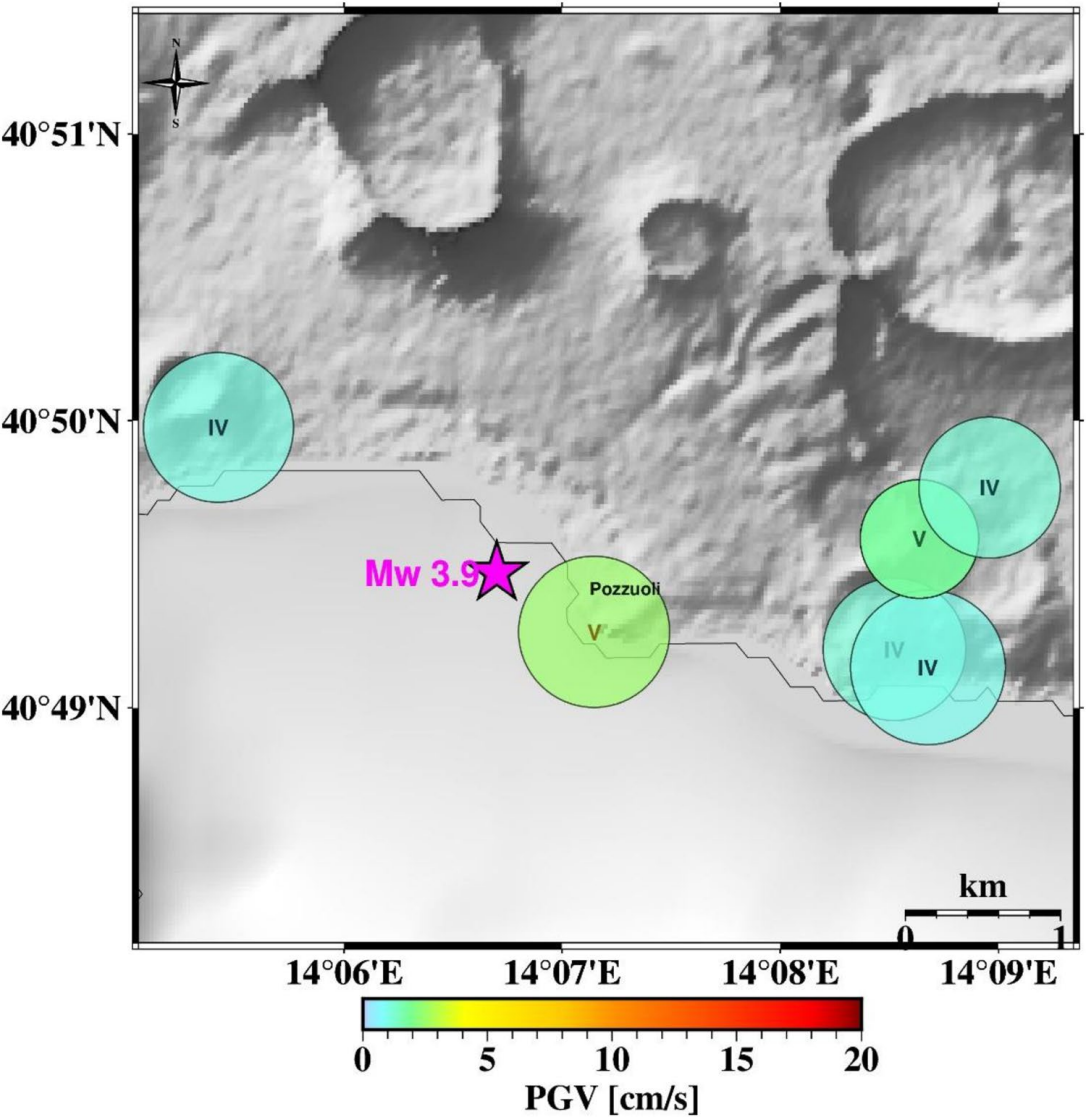
Test on the Md 4.6 earthquake of March 13, 2025. The map shows the result obtained in PTW = 1 s. Magenta star represents the earthquake location (the moment magnitude obtained from Md-MW scaling law by Iervolino et al. 2024 is also shown). Each circle represents the area centered on the station within which the predicted PGV varies between ± 50% of its value. Color follows PGV. In each circle, the estimated shaking intensity (IMCS) at the site is also reported.

In Fig. 4 and 5, we show the test on two earthquake scenarios, the Md 4.4 occurred on May 20, 2024 and the Md 4.6 occurred on March 13, 2025 respectively (see supplementary Table S1 for source parameters). These two events -among the largest registered in the area-  are not included in the dataset used for calibration and testing of the system.

For both scenarios, we computed the predicted impact area around each station within which the predicted PGV (PGV_pred_) may vary between  ±50% of its value. Since each single station can provide the expected PGV (PGV_pred_) and the magnitude (M_*EW*_) estimate, the couples (PGV_pred_ - 50%PGV_pred_, M_*EW*_) and (PGV_pred_ + 50%PGV_pred_, M_*EW*_) can be used to evaluate at first order the area within which this variation of the PGV will be experienced, using the local calibrated GMPE. Here, we use the GMPE from^21^, truncated to retain only the first term of hypocentral distance. In this way, each station defines its own “area of competence” (AoC hereinafter), that is the circular area around the station in which we expect values of predicted PGV ranging within a user-set percentage threshold.

## Discussion

The proposed methodology represents the first attempt to integrate early warning and rapid response strategies for small-to-moderate earthquakes, whose effects are typically confined to limited areas, a particularly challenging scenario for any early warning system. In this sense, the system is appropriately framed within an *Earthquake Early Response* paradigm, where information is delivered within seconds to tens of seconds after earthquake onset and is intended to support immediate situational awareness and short-term decision-making, rather than fully automated protective actions that require pre–S-wave alerts (e.g., elevator shutdown; rail and metro braking; gas and fuel line isolation; industrial process safe-mode activation.)^11,12^. This approach establishes a framework for low-latency, impact-based early response in contexts where small magnitudes, short source-to-site distances, and minimal lead times limit the effectiveness of conventional EEW applications. In the Campi Flegrei area, where seismicity often occurs close to buildings and critical infrastructure, traditional protective actions for individuals (e.g., “Drop, Cover, and Hold On”) are impractical, due to the very short warning times. Nevertheless, the proposed system is well suited to support early rapid response actions compatible with short latencies. Typical applications include the rapid activation of emergency management protocols, the prioritization of post-event inspections and response resources in areas expected to experience the strongest shaking, and the support of operational decisions at critical facilities — such as hospitals, industrial plants, or transportation hubs — where informed human intervention can still mitigate cascading impacts and enhance safety in the immediate aftermath of the event.

In this sense, a key aspect of our method is the quantification of the earthquake impact zone and its circular AoC around each station, where the expected PGV does not fall outside a selected range of values, which we set in our study within ± 50% of PGV predicted at the station site. In general, the choice of the percentage number depends on the intrinsic uncertainties related to the used GMPE (typically 1-sigma) and the expected extent of the affected area around the recording station. To address the issues of this parameter sensitivity, we added additional figures in the Supplementary Material (Supplementary Figures S5-S7) exploring different PGV variation thresholds. Larger PGV variations (e.g., ± 70%) result in average radii of competence of approximately 5–8 km, which are comparable with the extent of the entire studied area. Conversely, smaller PGV variations (e.g., ± 30%) correspond to smaller areas around the station, with average radii ranging between 0.5 and 2.5 km.

In the Campi Flegrei context, a ± 50% threshold represents a reasonable compromise between spatial coverage and variability of the ground motion prediction, given the small magnitudes and strong peak shaking attenuation typical of the area. More generally, the threshold can be considered a user-set parameter: depending on the size of the target to be protected and the type of safety actions to be activated, different PGV variation levels may be adopted.

The concept of AoC is intended to bridge the classical on-site EEW approach with the spatial extent of the expected damage zone. In a standard on-site EEW system, the information provided by a station refers strictly to the expected shaking at the installation point. However, from a physical perspective, this information is not confined to a single point but can be extrapolated, using region-specific GMPEs, over a surrounding area where the ground motion characteristics are expected to be confined within a range of the onsite predicted peak shaking. Therefore the AoC aims to quantify the spatial extent over which the information provided by a single station can be considered representative and therefore actionable.

From an application standpoint, users within the AoC may initiate mitigation actions depending on the target infrastructure or building and the required response time of emergency measures. For example, in the case of critical infrastructures such as hospitals, schools, or industrial facilities, the AoC may delineate the region within which automatic or semi-automatic protective actions can be safely activated on the basis of the on-site estimates. Beyond these areas, users may either adopt communication and response measures tailored to the expected level of ground shaking relative to the predicted peak-shaking range in the alerted zone, rely on information provided by other stations, or receive no alert at all.

The tests performed on the two scenario events show that although the system provides short lead-times, due to the proximity between the earthquake hypocenter and the station, it rapidly informs on the earthquake impact by predicting the peak ground shaking around the recording station (Fig. 4 and 5). A potential network of distributed single-station nodes, even without communication between them, would provide a realistic map of the earthquake impact across the area. In a sufficiently dense seismic network, areas not covered by on-site systems are expected to be limited. Where areas of competence associated with multiple stations overlap, a possible implementation would rely on a decision module capable of integrating and reconciling information from different on-site sensors. Although the method proposed here is inherently on-site, the integration of multiple on-site systems naturally moves toward a network-based, impact-oriented framework, consistent with approaches such as P-wave shaking-forecast methods (e.g.^18^) or S-wave propagation schemes based on local undamped motion (PLUM)^17^.

The methodology proposed here can be easily integrated into other platforms for alert dissemination and emergency management during natural, catastrophic events. As an example, the system could be integrated into a Multi-Risk Impact-based Early Warning Systems (MR-IEWS) within the framework of the seismic risk assessment. The MR-IEWSs aim at improving impact forecasting for geo and weather hazards, building accessible user-friendly platforms for the dissemination of high-resolution information at the occurrence of potential catastrophic events. (e.g. https://gobeyond-project.eu/ ).

A natural evolution of the proposed method is its direct connection to mobile apps or other devices (i.e., tablet, smartwatches) for the dissemination of earthquake alerts and immediate post-event information. Equipped with geolocation sensors, these devices can be used to track the position of people, before, during and after the occurrence of an earthquake, providing essential information to guide and prioritize rescue operations^22,23^. Providing immediate, reliable information right after an earthquake is extremely valuable for the population, for emergency management, and especially to counteract the potential spread of misinformation or fake news, effectively helping to reassure the public^24,25^.

Moreover, the developed methodology can be exported and adapted to other densely populated volcanic or seismically active regions worldwide, where conventional regional EEW systems face similar physical limitations. For application in a new region with more extended areas of impact, a specific calibration of parameters of the used empirical relations would be required. This would primarily involve (i) the selection or calibration of suitable local or regional/local GMPEs for the target area, (ii) the characterization of the typical source–station distance distributions based on the local seismic network geometry and seismicity patterns, (iii) assessment of expected uncertainties on output parameters, e.g. magnitude and predicted PGV. Importantly, these requirements rely on information that is commonly available in monitored regions and do not require changes to the core methodology. Once these elements are defined, the proposed framework can be applied using the same processing chain and decision logic presented in this study.

## Data & methods

### Waveforms preprocessing

The preliminary dataset consists of 524 earthquakes with duration magnitudes (Md) ranging from 1 to 4, which occurred in the Campi Flegrei caldera between January 1, 2016, and May 20, 2024, as reported by the ONT (Osservatorio Nazionale Terremoti, the Italian National Earthquake Observatory, https://terremoti.ingv.it) and GOSSIP, INGV-Oss. Vesuviano^26^. The original data were downloaded from the FDSN web service (waveforms available at http://webservices.ingv.it, last accessed on September 2025). Waveforms were recorded by the INGV-OV (Istituto Nazionale di Geofisica e Vulcanologia, Osservatorio Vesuviano section) seismic network, which includes 15 stations equipped with accelerometer and velocimeter sensors (represented as triangles in Fig. 1). The event locations were refined by Scotto di Uccio et al.(2024)^31^(see Fig. 1).

Prior to the system preprocessing, the dataset consisted in 3270 waveforms. To calibrate and test the system, we divided the dataset into two subsets: 80% of the waveforms (n = 2616) was used to calibrate the empirical scaling laws, while the remaining 20% (n = 654 waveforms) was used to test the system performance (see panel B of Fig. 1 for subsets distribution). The number of available waveforms for the final training and test datasets are summarized in Table 2.

**Table 2:** Total number of waveforms before and after the preprocessing phase of the analysis.

Dataset	Number of waveforms (raw)	Number of waveforms (after preprocessing)
Train	**2616**	**847**
Test	**654**	**448**
Total	**3270**	**1295**

### Calibration of empirical scaling laws, magnitude estimation and impact area evaluation

After the pre-processing, the following step was to calibrate the empirical scaling law that links the P-peak of displacement (Pd) to the Peak Ground Velocity (PGV)^27,28^.

We selected a 1-s P-wave time window (PTW) to measure Pd. To ensure data quality, we evaluated the theoretical S-wave arrival times and excluded waveforms that may have been contaminated by later phases.

Specifically, the initial peak displacement (Pd) and the frequency content (τc) are extracted from the vertical component of waveforms, while the observed PGV was computed as the maximum among horizontal components^27^. The resulting scaling law in PTW = 1 s was obtained from a linear regression on the calibration subset as:

$${\text{logPGV }} = {\text{ A}} + {\mathrm{BlogPd}}\left( {{\mathrm{PTW}} = {\mathrm{1s}}} \right){\text{ }} \pm {\mathrm{SE}}$$

where SE is the Standard Error.

For the magnitude estimate from a single station, here we propose and experimented a new methodology.

The method consists of the computation of the seismic moment M_0_ as the area underneath the source time function. Assuming a triangular shape for the source time function^28^, the seismic moment is computed as:

$${M}_{0}=\frac{4\pi {c}_{p}^{3}\langle R\rangle }{{F}_{s}{\mathcal{R}}_{\theta \varphi }}\frac{{P}{d}\cdot{\tau }_{c}}{2}$$

where $\rho$, c_p_, F_s_, ${\mathfrak{R}}_{\theta \varphi }$ are fixed. Pd is assumed to be the peak of the source time function with the duration of ${\tau }_{c}$. The τ*c* is the characteristic period of the chosen P-signal and it is computed according to the approach of^29^ in a time window, PTW = 1 s. Here we use the properties that τ*c* is equal to the reciprocal of the corner frequency^30^ and it approximates the rupture duration, < R > is the mean hypocentral distance of the distances distribution of each station in the area. The seismic moment is then converted to moment magnitude.

Finally, we evaluated the impacted area around the station where the predicted PGV varies within ± 50% of its value, called PGV_pred150_ and PGV_pred50_ respectively. Indeed, combining the magnitude estimate and the predicted PGV at the site with the GMPEs in the area (Scala et al. 2025), we can evaluate Rmin and Rmax as it follows:

$$\mathrm{log}{R}_{min} = \frac{\mathrm{log}{PGV}_{pred 150}-{A}^{{\prime}{\prime}}-{B}^{{\prime}{\prime}}{M}_{pred}}{{C}^{{\prime}{\prime}}}$$

$$\mathrm{log}{R}_{max}=\frac{\mathrm{log}{PGV}_{pred 50}-{A}^{{\prime}{\prime}}-{B}^{{\prime}{\prime}}{M}_{pred}}{{C}^{{\prime}{\prime}}}$$

where A’’, B’’, C’’ are the coefficients of the GMPEs of the area of interest. The difference ΔR (R_max_-R_min_)/2 represents the radius of the impacted area around the station. In Supplementarty Figure S5-S7 we showed expected values for ΔR in the Campi Flegrei area at different ranges of magnitude and PGV.

### System performance assessment and impacted area evaluation

The performance of the system is evaluated in terms of:

Successful Alert (SA): IMCS_pred_ ≥ IMCS_threshold_ & IMCS_obs_ ≥ IMCS_threshold_.

Successful No-Alert (SNA): IMCS_pred_ $<$ IMCS_threshold_ & IMCS_obs_ $<$ IMCS_threshold_.

False Alert (FA): IMCS_pred_ $\ge$ IMCS_threshold_ & IMCS_obs_ < IMCS_threshold_.

Missed Alert (MA): IMCS_pred_ < IMCS_threshold_ & IMCS_obs_ $\ge$ IMCS_threshold_.

where IMCS_pred_ is predicted by the system and IMCS_threshold_ is a threshold value which potential users can set. We quantify the performance in terms of precision and recall, defined as^10^:

$$Precision= \frac{SA}{SA+FA}$$

$$Recall= \frac{SA}{SA+MA}$$

## Supplementary Information

Supplementary Information.

## Data Availability

The data analyzed in this study were obtained from Istituto Nazionale di Geofisica e Vulcanologia via the FDSN web service (open access: https://terremoti.ingv.it/webservices_and_software; last accessed September 2025).
