# Supplementary material for: One-second-lead earthquake warning and impact assessment at Campi Flegrei
Source: Sci Rep. 2026 Apr 8;16:16630. doi: 10.1038/s41598-026-42593-x (PMC13219473; doi:10.1038/s41598-026-42593-x)
Supplement: Supplementary file 1 — Supplementary Information. [file 41598_2026_42593_MOESM1_ESM.docx]

*Supplementary material of*

**One-Second-Lead Earthquake Warning and Impact Assessment at Campi Flegrei**

*V. Longobardi ^1^, S. Colombelli^1^ and A. Zollo^1^**

^1^ Department of Physics “Ettore Pancini”, University of Naples “Federico II”, Naples, Italy

*corresponding author (email: [aldo.zollo@unina.it](mailto:aldo.zollo@unina.it))


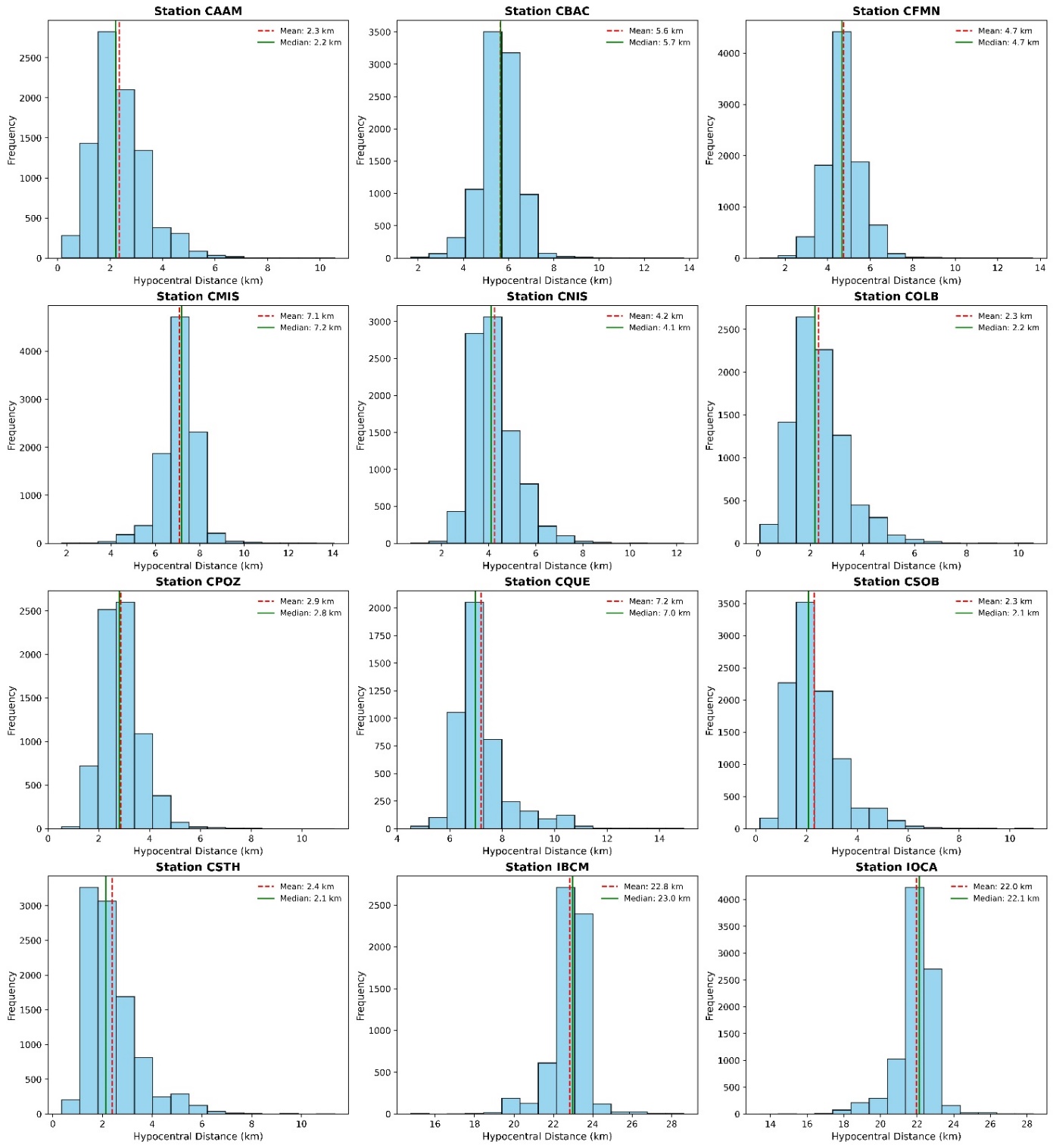
Figure S1: Hypocentral distances distrubutions for stations in the area. Each histogram shows the station code in the title. Mean and median of each distribution are shown with a vertical red dashed line and solid green line respectively.


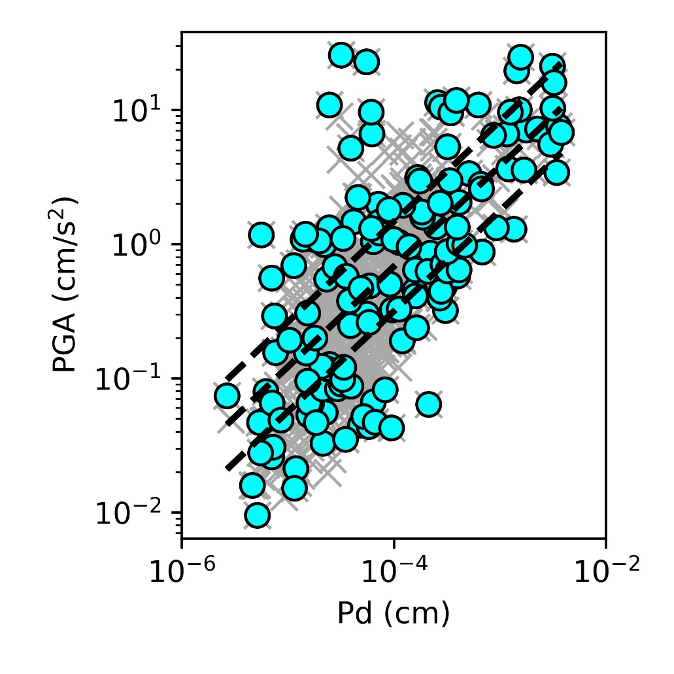


Figure S2 shows the scaling of PGA vs PD in a one-second P-time window. Dark grey crosses represent single station measurements on the train dataset. Cyan points represent 2-d binned data (x-bin width = 1 cm and y-bin width = 0.5 cm/s). Dashed lines are the calibrated PGA vs Pd law and its standard error bounds for the 2-d binned data.


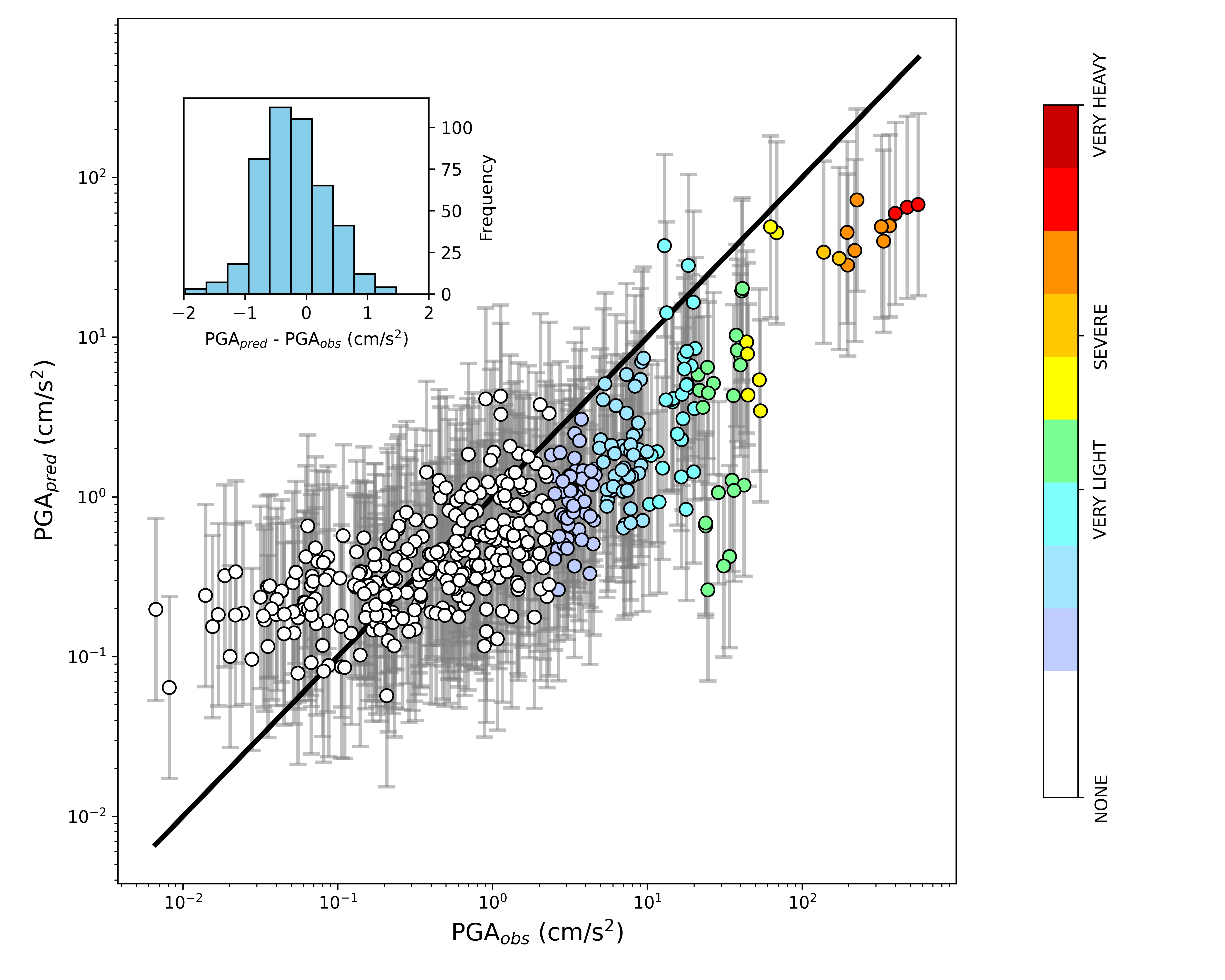
Figure S3 shows the predicted PGA of the test dataset with respect to the observed PGA, using the calibrated law of PGA vs Pd in one-second P-time window. Points are colored following the expected potential damage scale. The black solid line is the one-to-one line. In top left corner, the histogram of the prediction error (PGApred – PGAobs) is reported.


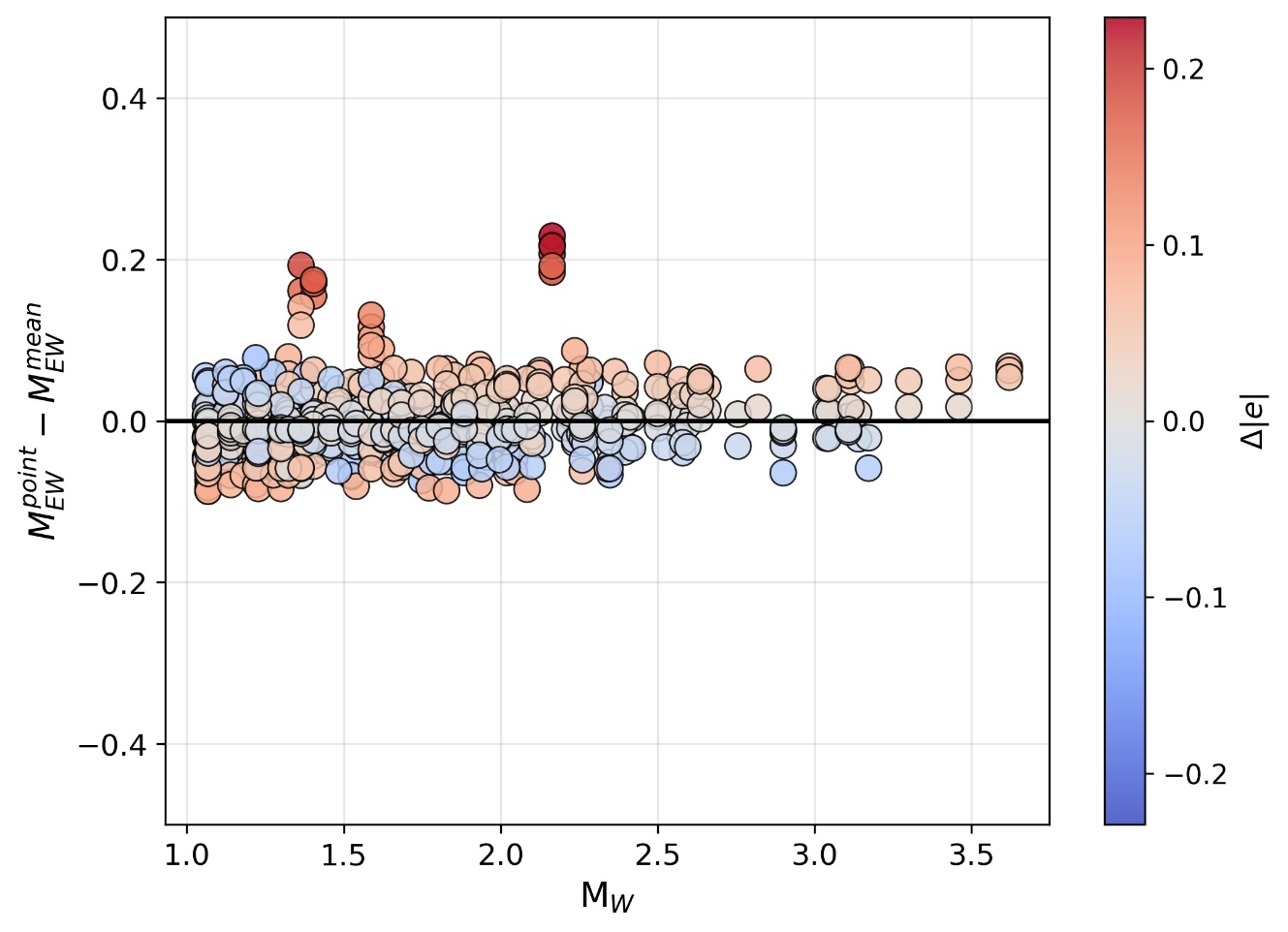


Figure S4: Comparison between early-warning magnitude estimates computed using station-specific mean hypocentral distance and point hypocentral distance. The plot shows the difference between the two estimates (M${point}_{\text{EW}}$ − M${mean}_{\text{EW}}$) as a function of the reference moment magnitude (Mw), isolating the effect of the hypocentral distance parameterization.

The colorbar follows the difference in absolute error with respect to Mw, defined as
$\Delta\mid e\mid=\mid MEW_{\text{mean}}-M_{\text{W}}\mid-\mid MEW_{\text{point}}-M_{\text{W}}\mid$.
Positive values of $\Delta\mid e\mid$indicate cases in which the use of point hypocentral distance yields magnitude estimates closer to Mw than those obtained using station mean hypocentral distance.


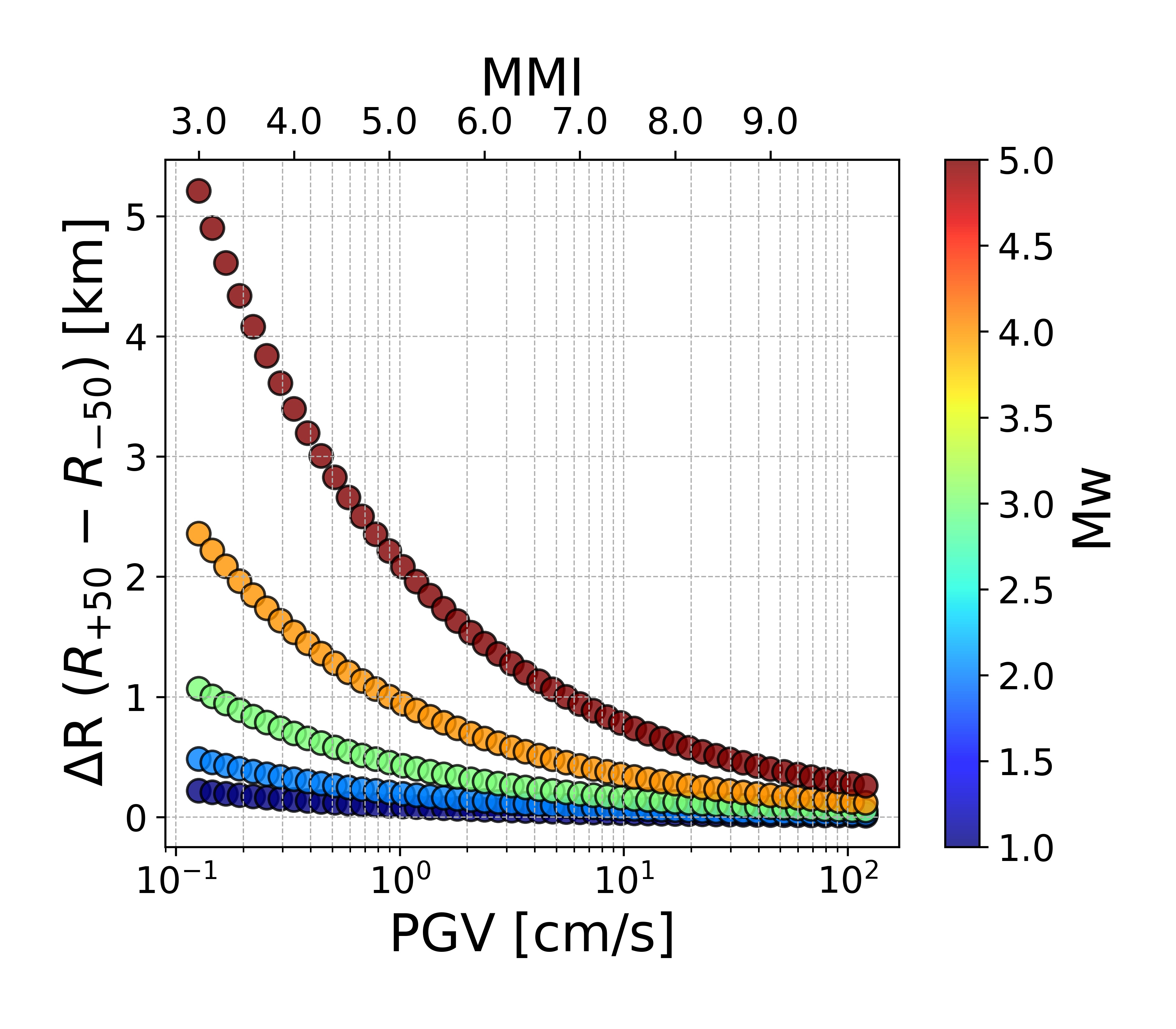


Figure S5 represents the radius ΔR (R_max_-R_min_) of the impacted area versus PGV and moment magnitude Mw. Within the impacted area, the PGV is expected to vary within ±50% of its value. On secondary y-axis the corresponding value of intensity is also shown.


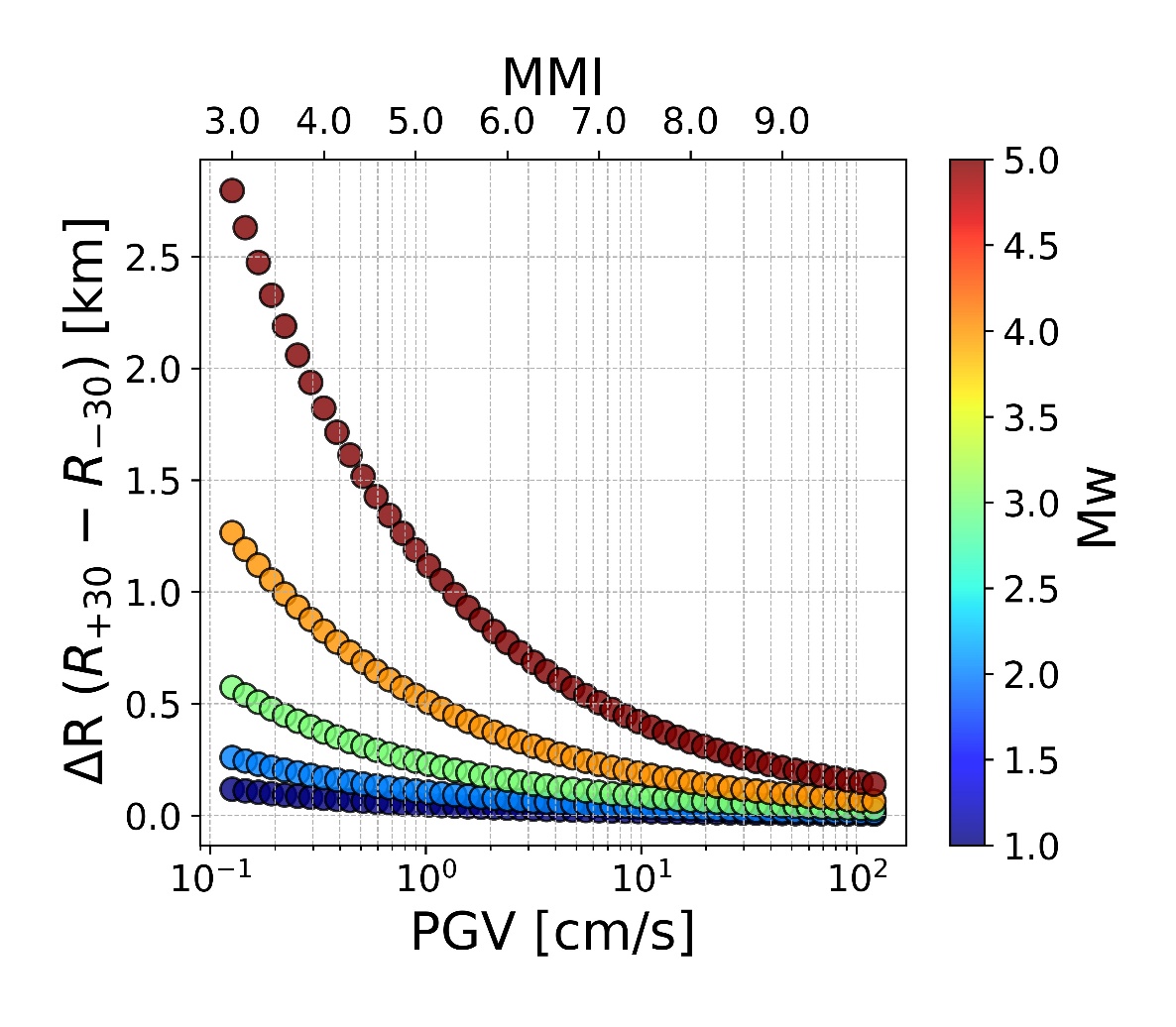


Figure S6 represents the radius ΔR (R_max_-R_min_) of the impacted area versus PGV and moment magnitude Mw. Within the impacted area, the PGV is expected to vary within ±30% of its value. On secondary y-axis the corresponding value of intensity is also shown.


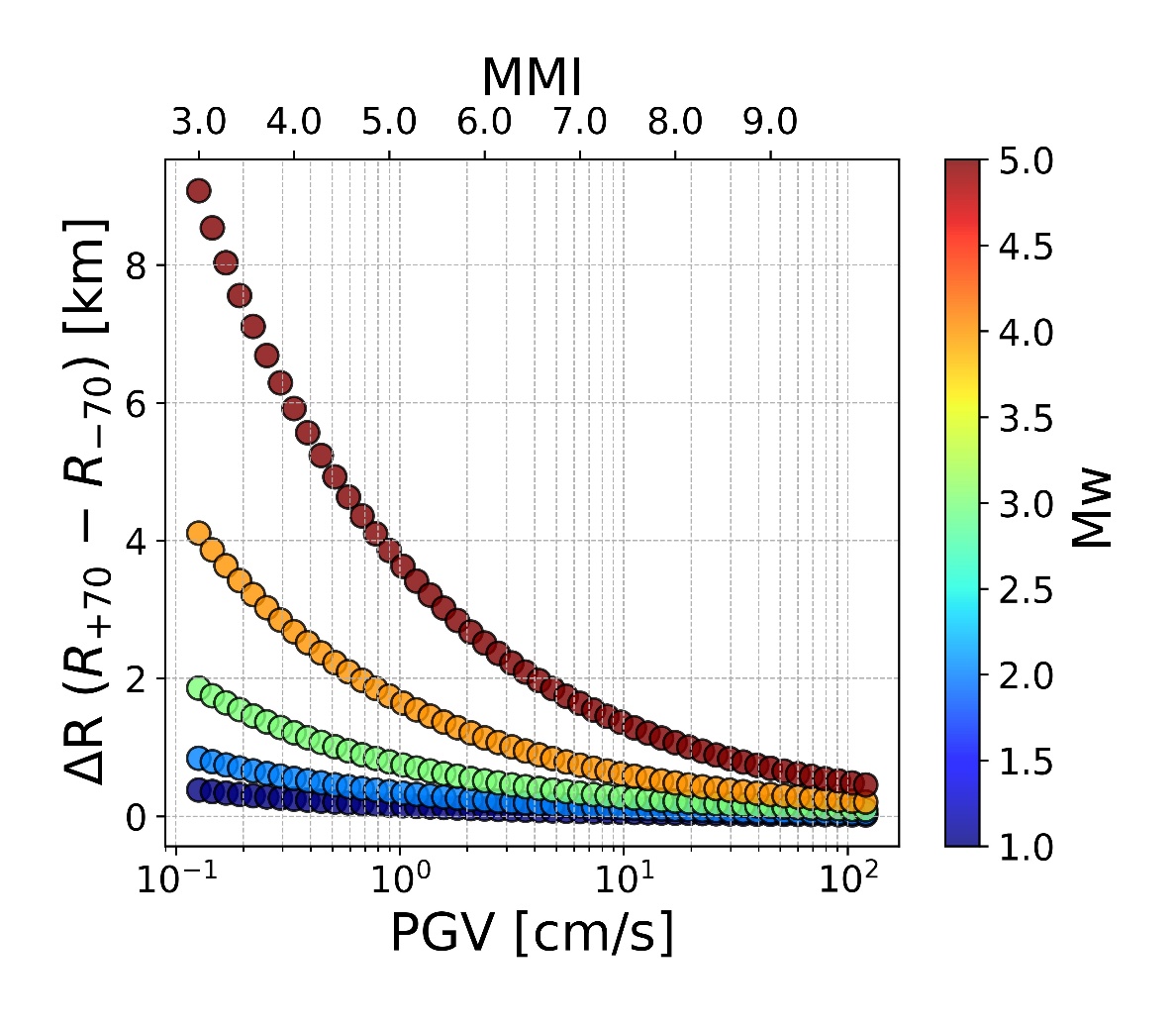


Figure S7 represents the radius ΔR (R_max_-R_min_) of the impacted area versus PGV and moment magnitude Mw. Within the impacted area, the PGV is expected to vary within ±70% of its value. On secondary y-axis the corresponding value of intensity is also shown.

Table S1: Source parameters of the two earthquake scenarios

| Origin time (UTC) | Latitude (°N) | Longitude (°E) | Depth (km) | Magnitude (Md) | Magnitude (Mw) |
| --- | --- | --- | --- | --- | --- |
| 2024-05-20 18:10:03.69 | 40.8250 | 14.1391 | 2.20 | 4.4 | 3.8 |
| 2025-03-13 10:07:34.00 | 40.8245 | 14.1117 | 2.63 | 4.6 | 3.9 |
